# Environmental control for X-ray nanotomography

**DOI:** 10.1107/S1600577522006968

**Published:** 2022-07-21

**Authors:** Mirko Holler, Tomas Aidukas, Lars Heller, Christian Appel, Nicholas W. Phillips, Elisabeth Müller-Gubler, Manuel Guizar-Sicairos, Jörg Raabe, Johannes Ihli

**Affiliations:** a Paul Scherrer Institut, Forschungsstrasse 111, Villigen PSI, Aargau 5232, Switzerland; Uppsala University, Sweden

**Keywords:** *in situ* nano-tomography, ptychographic tomography, ptychography, environmental control

## Abstract

The acquisition speed and spatial resolution of X-ray nanotomography have continuously improved over the last decades. Coherent diffraction-based techniques breach the 10 nm resolution barrier frequently and thus pose stringent demands on sample positioning accuracy and stability. At the same time there is an increasing desire to accommodate *in situ* or *operando* measurements. Here, an environmental control system for X-ray nanotomography is introduced to regulate the temperature of a sample from room temperature up to 850°C in a controlled atmospheric composition. The system allows for a 360° sample rotation, permitting tomographic studies *in situ* or *operando* free of missing wedge constraints. The system is implemented and available at the flOMNI microscope at the Swiss Light Source. In addition to the environmental control system itself, the related modifications of flOMNI are described. Tomographic measurements of a nanoporous gold sample at 50°C and 600°C at a resolution of sub-20 nm demonstrate the performance of the device.

## Abstract 2

An environmental control system for *in situ* or *operando* nanotomography is presented. It allows controlling the sample temperature from room temperature to 850°C under controlled gas compositions, and a 3D isotropic resolution of sub-20 nm at a temperature of 600°C is demonstrated.

## 1. Introduction

X-ray nanotomography is developing from a niche research area to a centerpiece of modern material and process characterization studies. This transformation is driven among other factors by faster acquisition times and improved spatial resolution. Selected X-ray imaging modalities and associated tomography techniques are now able to reach the 10 nm resolution range reliably (Shapiro *et al.*, 2020 ▸; Holler *et al.*, 2014 ▸, 2019 ▸; Rösner *et al.*, 2020 ▸).

In particular, coherent X-ray diffraction imaging based techniques (Miao *et al.*, 2015 ▸) benefit strongly from the just completed and upcoming upgrades of synchrotron light sources (Eriksson *et al.*, 2008 ▸). These methods will allow us to reduce the resolution gap to electron microscopy, while examining representative sample volumes (Ross & Minor, 2019 ▸; Taheri *et al.*, 2016 ▸). With the improvements in spatial resolution and acquisition time, we are increasingly confronted with the demand for environmental X-ray nanotomography capabilities. This is to examine functional materials and processes under conditions that are representative of their real application (Beck *et al.*, 2021 ▸; Meirer & Weckhuysen, 2018 ▸; Wood, 2018 ▸).

Environmental setups that facilitate *in situ* and *operando* studies of materials in response to changes in atmosphere, temperature and/or pressure are a hallmark of X-ray microtomography (Zhu *et al.*, 2021 ▸; Maire *et al.*, 2001 ▸; Marone *et al.*, 2020 ▸; Meirer & Weckhuysen, 2018 ▸; Wood, 2018 ▸). However, equivalents for similar studies at nanometric 3D resolution are rare (Weber *et al.*, 2022 ▸; Watanabe *et al.*, 2007 ▸; Murata *et al.*, 2015 ▸) because of the more stringent requirements concerning sample stability, positioning accuracy and angular sampling, especially in scanning-based imaging modalities (Pfeiffer, 2018 ▸; Mokso *et al.*, 2007 ▸). Artifact-free tomographic reconstructions with isotropic resolution require the acquistion of angularly equally spaced projections covering the full angular span from 0° to 180°. The number of projections is thereby set by the Crowther criterion and scales, for a fixed sample thickness, inversely to the target spatial resolution (Crowther *et al.*, 1970 ▸). If the angular sampling range is reduced, the data are incomplete leading to a missing wedge in Fourier space and thus non-isotropic 3D resolution and reconstruction artifacts.

The few existing environmental control nanotomography setups follow the design of environmental transmission electron microscopy cells (Fam *et al.*, 2019 ▸; Weber *et al.*, 2022 ▸; Kahnt *et al.*, 2021 ▸; Wu *et al.*, 2017 ▸; Parker *et al.*, 2022 ▸). These delimited cells are equipped with imaging windows and constrain the angular sampling range. Additionally, the imaging thickness is angle dependent. The latter can be circumvented by using lamino­graphy (Holler, Odstrcil *et al.*, 2020 ▸; Holler *et al.*, 2019 ▸; Donnelly *et al.*, 2020 ▸), still having the disadvantage of missing information resulting in artifacts and loss of quantitativeness, which might be important for chemical characterization. Another issue is related to the mass of some cells which in scanning imaging modalities need to be physically moved during acquisition. This can reduce the sample scanning speed and precision.

We present an alternative approach of an environmental control system for X-ray nanotomography which circumvents the missing wedge problem and allows quantitative contrast at 3D isotropic resolution. The system was implemented in the flexible tOMography Nano Imaging microscope (flOMNI) at the Swiss Light Source (SLS) (Holler *et al.*, 2012 ▸, 2014 ▸). Positioning accuracy in flOMNI is achieved by a dedicated interferometry feedback system (Holler & Raabe, 2015 ▸). The environmental control is based on the delivery of a controlled and conditioned gas stream to a sample holder which is engineered for minimal heat transfer. The target gas or gas mixture required for *in situ* or *operando* studies can be heated up to 850°C. Instrument-compatible gases are evaluated on a case-by-case basis, with reference to the safety regulations of the institution and secondary experimental parameters. For example, even the use of flammable and corrosive gases, such as H_2_, is possible, when used in the form of a forming gas or within a safe temperature range. Environmental nanotomography capabilities are demonstrated via ptychographic X-ray computed tomography (PXCT) (Pfeiffer, 2018 ▸) of a nanoporous gold sample at contrasting temperatures under N_2_ gas flow. Measurements revealed the presence of marginal sample drifts across the target temperature window. Fourier shell correlation analysis suggests that the corresponding phase or electron density tomograms possess a sub-20 nm spatial resolution regardless of the operation temperature. We emphasize that the concept can easily be implemented in other microscopes, acquisition and imaging modalities.

## 2. The heating nozzle

The current sample scanning accuracy of flOMNI is in the sub-10 nm range. At SLS it is predominantly used for tomography of samples up to 150 µm in diameter with nanometric spatial resolution in the tender and hard X-ray regime, *i.e.* from 4.7 to 12.4 keV (Holler, Guizar-Sicairos *et al.*, 2017 ▸; De Angelis *et al.*, 2017 ▸; Lin *et al.*, 2020 ▸). The acquisition of a single projection via X-ray ptychography can be as short as 10 s, but can also require up to 120 s depending on the experimental requirements. To not compromise the instrument’s performance, any introduced environment control system should not increase sample positioning errors of flOMNI within the single projection measurement time scale.

The presented environmental control system achieves this by mechanically decoupling the sample positioning instrumentation from the heat source. The sample is heated indirectly via a thermally controlled target gas. The nozzle encloses the sample, creating a localized hot zone of constant atmospheric composition confined to the sample region. Gas flow escaping the sample region is captured by an integrated gas exhaust system. To minimize the heat transfer to the instrument, as well as air density fluctuations, which would interfere with the interferometric position metrology, the outer shell of the heater is cooled and the temperatures of critical setup components are locally controlled.

**Figure 1:**
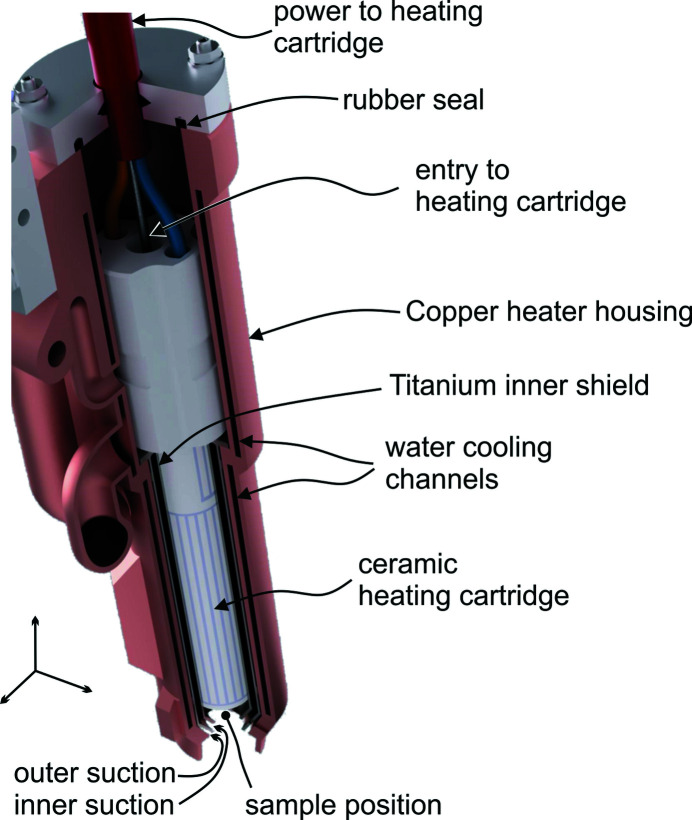
3D rendering of the heater nozzle. Arrows of the coordinate system correspond to 1 cm length.

Fig. 1 ▸ provides a 3D rendering of the nozzle detailing its structural organization and components. The core of the heating device comprises a commercial ceramic heating cartridge (800001463 Lamellenheizer, Paul Rauschert Steinbach GmbH) providing power up to 165 W. The cartridge is hollow on the inside and the gas flow enters through its back. The gas flow rate is measured by a flow meter (SFTE-2U-V, Festo SE & Co. KG). After leaving the heater, the high-temperature gas is brought into contact with the sample. For measuring the gas temperature close to the sample position a thermocouple probe (K type, TJ36-CAIN-116U-6-CC-XCIB, Omega Inc.) is placed just above the gas outlet of the cartridge. The exhaust system is driven by a Venturi nozzle (VN-05-L-T3-PQ2-VQ2-RO1, Festo SE & Co. KG). This nozzle is operated by pressurized air and the suction rate is measured by a flow meter. Under typical flow rate settings, *i.e.* between 1 and 2 l min^−1^, no disturbance of the laser interferometry of the sample position metrology is observed. This indicates that all gas provided by the heating nozzle is removed by the exhaust.

**Figure 2:**
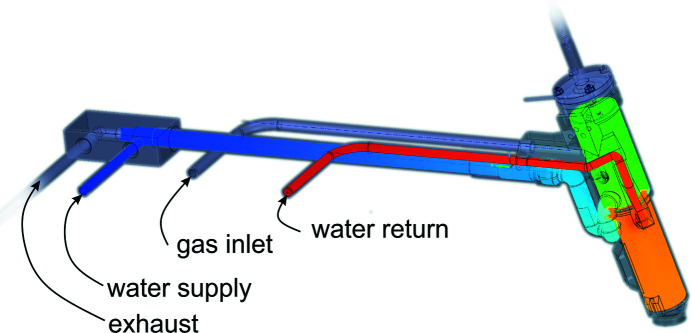
Cooling and gas connections of the heater nozzle. The gas exhaust pipe is surrounded by the cooling water supply (blue), which enters the upper region of the nozzle (green) first, then flows to the lower region where most heat is to be removed (orange) and then leaves the nozzle (red).

The amount of power and thus temperature provided by the heater can be significant. The heating cartridge is therefore isolated by a multi-layered enclosure. The inner two layers are formed by the gas flow of the exhaust system, the outer layer by an active water-cooling. This configuration not only immediately cools the exhaust gas but also ensures that the outer shell of the housing remains close to room temperature even at full power. Fig. 2 ▸ shows how gas flow, exhaust and water-cooling are connected to the nozzle.

Given the complex structure of the 1 mm-wide flow channels for gas and cooling water, the nozzle could not be manufactured by conventional machining and was 3D printed from copper. The pipe connections are fixed to the heater enclosure by ep­oxy adhesive (ND353, EPO-TEK Inc.).

## 3. Modifications to the tomography instrument

The introduction of the environmental control system, in particular of a potentially high-temperature and corrosive gas stream, necessitates appropriate modifications of the X-ray tomography instrumentation, here the flOMNI microscope (Holler *et al.*, 2012 ▸, 2014 ▸). Two of its components are in contact with the gas stream. These are the sample pin holder and the order-sorting aperture (OSA) which can approach the sample down to a distance of 0.5 mm. Modifications of the sample holder and OSA are discussed separately in detail below.

**Figure 3:**
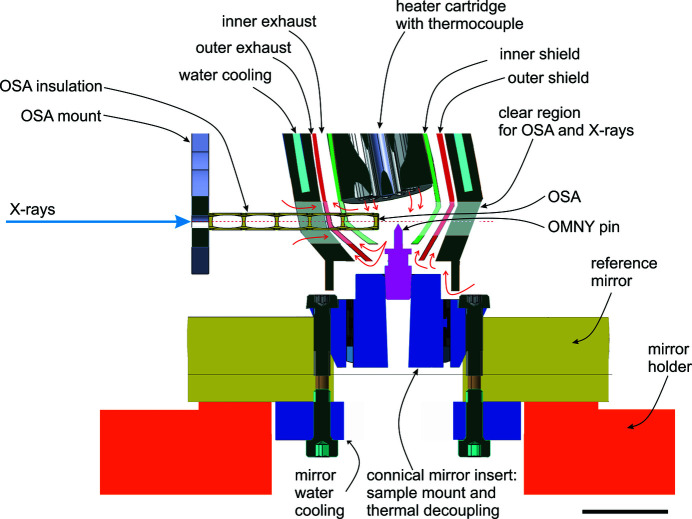
Vertical cut, along the X-ray propagation direction, through the setup at the sample plane. The red arrows in the nozzle region indicate the gas flow directions. Scale bar: 10 mm.

Fig. 3 ▸ displays a vertical cut through the setup at the sample plane along the X-ray propagation direction. Black arrows are used to label individual components. Red arrows indicate the gas inflow and exhaust directions. The gas exhaust flow-rate is by default set to be five times larger than the gas inflow-rate. The additional volume is drawn from the instrument-surrounding atmosphere. This arrangement is rooted in the open nature of the environmental control system and safety concerns. The setup geometry shall ensure the sample is subjected only to a target gas of constant composition and temperature, yet the higher exhaust flow-rate shall ensure that gases or reaction products that are potentially damaging to health or instrumentation are immediately diluted, cooled down and removed from the system, all while not limiting the angular resolution nor the quality of the acquired tomograms.

### 3.1. Sample holder and mirror

The sample stage in flOMNI consists of a sample pin positioned on a reflective mirror for interferometric position measurement. The reference mirror is unchanged compared with earlier versions of flOMNI. It has a conical receptor into which an adaptor for the tomography pin can be mounted (conical mirror insert in Fig. 3 ▸). This flexibility allows, for example, installing sample holders for dual-axis tomographic measurements (Donnelly *et al.*, 2017 ▸).

**Figure 4:**
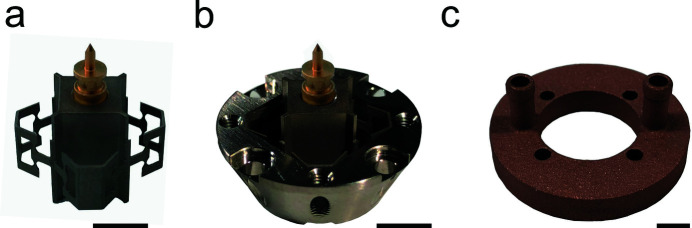
Modifications of the flOMNI sample holder. (*a*) An OMNY sample pin inserted into the new pin receptor with flexure structures designed to minimize heat transfer and mechanical stress to the laser interferometer mirror. (*b*) Receptor mounted in the conical mirror mounting piece. (*c*) 3D printed water cooler mounted to the back of the laser interferometer mirror. Scale bars: 5 mm.

To ensure stable and accurate sample scanning, the mirror must maintain its shape and dimensions throughout the heating cycle, for which a new mirror insert was designed. In consideration of the different materials of sample pin (aluminium, ceramics or copper), pin receptor (titanium) and mirror (aluminium) and associated differences in thermal expansion and heat conductivity, the structure was designed to minimize mechanical stress and heat flow to the mirror. Fig. 4 ▸(*a*) shows the new pin receptor with a regular OMNY pin (Holler, Raabe *et al.*, 2017 ▸). It is connected to the conical mount of the mirror via a stiff flexure structure. Fig. 4 ▸(*b*) shows this flexure structure mounted using ep­oxy adhesive (T7110, EPO TEK Inc.) to the conical mounting piece. The new mirror insert is manufactured from titanium.

Finally, to ensure a stable reference mirror temperature we designed and mounted a 3D printed water cooler [Fig. 4 ▸(*c*)] to the bottom of the mirror (mirror water cooling in Fig. 3 ▸). For accurate temperature control, the mirror was further equipped with a temperature sensor and two micro-heaters.

In combination, these features ensure that the laser mirror shape and dimensions remain constant even at elevated temperatures, thereby facilitating at high temperatures the accurate sample scanning required for ptychographic nanotomography. In consideration of the mirror and sample holder design, horizontal drifts are expected to be zero. As the thermal expansion of the sample pin and holder are not directly compensated, the sample position in the vertical direction is expected to change in response to temperature changes. For measurements with continuous temperature ramps we have implemented a feedback loop based on image registration. More details on this are given in Section 6.

### 3.2. OSA holder

In flOMNI, beam-shaping and focusing onto the sample is achieved by a gold Fresnel zone plate (FZP) with engineered optical aberrations (Odstrcil, Lebugle, Lachat *et al.*, 2019 ▸). A combination of a 50 µm central stop (CS) integrated in the FZP and a 30 µm OSA are used to block the non-diffracted part of the X-ray beam as well as higher and negative diffraction orders. To avoid the OSA clipping the first-order scattering signal, having a beam size at the OSA below the 20 µm range is desirable, leading to an OSA-to-sample distance in the 1 mm range. This has the consequence that the OSA will be subjected to the incoming (hot) gas stream and has approximately the same temperature as the sample. The acceptable drift of this setup component in the plane perpendicular to the X-ray propagation direction is in the range of a few micrometres.

**Figure 5:**
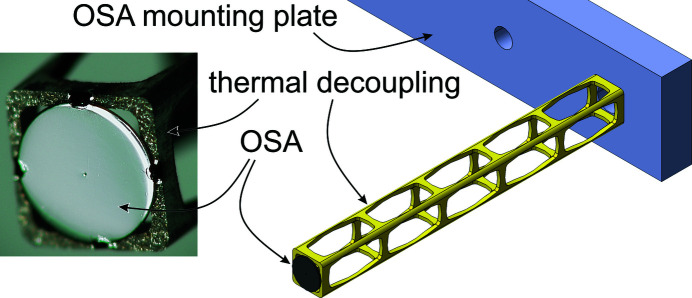
Left: micrograph of the designed OSA having an outer dimeter of 1.65 mm and holder. Right: the OSA is welded to a wire-eroded thermal decoupling structure (yellow) fixed on a temperature-controlled mounting plate.

To be able to operate in this positioning window we designed a new OSA mount and thermal decoupling structure, which attaches to the XYZ OSA positioning stages, shown in Fig. 5 ▸. The OSA is electron beam welded to the wire-eroded titanium structure. The OSA has an outer diameter of 1.65 mm and is manufactured from platinum. The thin beams of the isolation structure have a width down to 0.2 mm. The introduction of this structure allows a thermal decoupling of the (hot) OSA from the (room-temperature) mounting plate. The structure is aligned parallel to the beam direction such that its thermal expansion changes the position of the OSA along the X-ray propagation direction only. The mounting plate has further an active temperature control.

**Figure 6:**
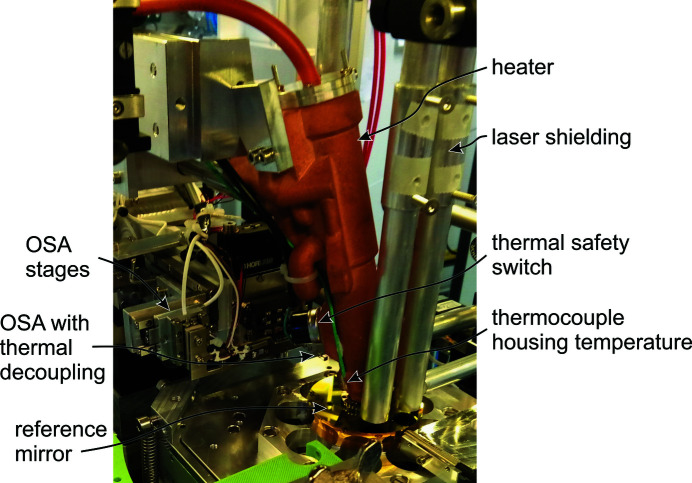
Photograph of all components as installed in flOMNI. The heater is moved down to the measurement position; the OSA has not approached yet and is outside the heater.

Fig. 6 ▸ shows a photograph of the heater mounted and in measurement position in flOMNI. The heater is installed on a motorized stage and can be moved up for sample change. In the photograph, the OSA is not yet inserted into the heater.

## 4. The control system and safety mechanisms

Four temperature controllers (CN16DPT-305-EIP-DC, OMEGA Inc.) are used to measure and control the temperature of various setup components. Temperature controller 1 is assigned to the regulation of the heating cartridge and is operated in closed-loop with a thermocouple probe (K type, TJ36-CAIN-116U-6-CC-XCIB, Omega Inc.). The probe is inserted from the back of the cartridge and ends flush directly above the OMNY tomography pin (Fig. 3 ▸). The heater cartridge is powered directly from a 230 V power line and is connected via a 10 mA fault current protection. A standard light dimming module (15.11.8.230.0400, Finder GmbH) is used for output regulation. The dimmer module has a 0–10 V voltage control input and is thus able to connect to the analog PID output of the temperature controller. This combination allows for a stable temperature control across the targeted temperature range, with remaining oscillations of ±1°C. See Fig. 8 for a demonstration. For cases where the temperature change is larger than 50°C, the set temperature is gradually increased to avoid overshooting. The heating cartridge, however, allows for temperature ramp rates of 15°C s^−1^, for temperatures below 400°C, decreasing gradually to marginal rates in the 10°C min^−1^ range when approaching a temperature of 850°C. The cooling rates are similar but display the reverse behavior, meaning large at high temperature and flattening towards room temperature. For safety reasons, the temperature controller remains in a wait status after power on and requires manual enabling, such that in the case of a power supply failure the setup will remain in an off state.

Temperature controller 2 is used to measure the temperature of the heater housing. The corresponding thermocouple (K type, 5LSC-GG-KI-24-1M) is glued to the outside of the housing using a thermally conductive ep­oxy (T7110, EPO-TEK Inc.). This controller is used for observation purposes only and disables the power to the heating cartridge in case of temperatures >30°C, which would indicate a cooling failure.

Temperature controller 3 measures the temperature of the laser reference mirror using a PT100 sensor (Pt 100C, Heraeus Sensor Technology) via a four-wire connection going through the center holes of the rotation and piezo stage of flOMNI. The analog PID output is used in conjunction with a power operational amplifier (L165, STMicroelectronics; for a schematic of a single channel, see Fig. S1 of the supporting information) to drive two heating elements (Pt 6,8 M 1080, Heraeus Sensor Technology), mounted on opposite sides of the mirror for better heat distribution. The heaters are connected in series and limited to a total of 2 W in heating power. The chiller (T257P, Thermotec Inc.) used for cooling the laser system of the interferometer is also used for cooling the mirror [Figs. 3 ▸ and 4 ▸(*c*)]. The temperature set-point of controller 3 is 0.5°C higher than the measured open-loop temperature. This arrangement allows for a stable temperature of the mirror (±0.01°C) over the entire targeted sample or heater temperature range, and thus minimizes thermal sample drifts.

Finally, temperature controller 4 akin to controller 3 measures and stabilizes the arm of the OSA holder, although in practice it has turned out that it is not required as the temperature of this component is stable even without active control.

In terms of system safety, it is ensured that the heating cartridge will switch off in the case of a cooling failure. An active safety mechanism is provided by temperature controller 2 as mentioned earlier. In addition, there is a passive temperature switch mounted at the heater housing with a switching temperature of 40°C as a second safety mechanism (Fig. 6 ▸).

The supply gas and exhaust flow rates are passively set by needle valves. Their flow is measured by electronic flow meters (SFTE-2U-V for the supply gas covering a measurement range from 0 to 2 l min^−1^, and SFTE-10U-V for the exhaust covering 0 to 10 l min^−1^, Festo SE & Co. KG) providing an analog voltage to a programmable logic controller (PLC 47300-16Bit, Galil Motion Control). This allows for remote monitoring of the flow rates and disabling the heater in case of gas supply or exhaust failure.

The cSAXS beamline uses *SPEC* (Certified Scientific Software, https://www.certif.com/) for experiment control. To interface the temperature controllers as well as the PLC, a softIOC provides several *EPICS* (*Experimental Physics and Industrial Control System*, http://www.aps.anl.gov/epics/) channels that can be accessed for control and measurement. A Python script is used for device communication with the temperature controllers and PLC.

## 5. Sample mounting

Sample stability and purity are a strict requirement for *in situ* or *operando* nanotomography. The selection of the sample-mounting medium and preparation technique is accordingly of increased importance. For brevity purposes, we consider the two dominant forms of sample preparation mounting techniques. (1) Focused ion-beam (FIB) milling – the transfer of the prepared sample cylinder and its fixation on the tomography pin using metal or carbon deposition (Furlan *et al.*, 2018 ▸). This method is preferable, albeit quite costly, as the chances of introducing impurities can largely be eliminated using modern milling systems. The choice of the type of metal or carbon for sample fixation purposes requires a case-by-case consideration. For example, the metal should not interact or form an alloy with the pin material and or the sample at elevated temperatures. Further, the amount of deposited metal and carbon should be minimized; this is to avoid the potential of thermal dewetting and subsequent sample contamination or, for example, of the clogging of porous sample materials. (2) The direct fixation of suitably size samples on the pin using adhesives (Holler, Ihli *et al.*, 2020 ▸). Equally of concern are environmental stability considerations and ideally an initially low adhesive viscosity, for example, as found in cement and carbon-based adhesives.

## 6. Nanotomography demonstration

To demonstrate the environmental nanotomography capabilities, we performed two PXCT measurements of a nanoporous gold sample (Larsson *et al.*, 2019 ▸) at the cSAXS beamline of SLS. Measurements were conducted at nominal temperatures of 50°C and 600°C under constant nitro­gen flow (1 l min^−1^).

The nanoporous gold sample was prepared via a de-alloying process using an aqueous nitric acid solution (Zinchenko *et al.*, 2013 ▸). A tomography pillar, 6 µm in diameter, was produced using a micro-lathe (Holler, Ihli *et al.*, 2020 ▸) and a focused ion-beam milling system. The sample pillar was annealed at 600°C prior to the acquisition of both tomograms. Annealing at the upper temperature allowed us to eliminate sample dynamics, *e.g.* the coarsening of the pore network (Chen-Wiegart *et al.*, 2012 ▸). This ensures a fair measurement comparison in terms of positioning and sample stability at low and high temperature. Future experiments will aim to study coarsening dynamics with nanoscale resolution (De Angelis *et al.*, 2017 ▸; Furlan *et al.*, 2018 ▸; Lin *et al.*, 2020 ▸). The pore diameter in the nanoporous gold sample was determined to be ∼10 nm on average using electron microscopy, shown in Fig. S2. Further details concerning the sample and its preparation are provided in the supporting information.

To acquire ptychography projections, the sample was coherently illuminated with a photon energy of 6.2 keV using an FZP with engineered optical aberrations (Odstrcil, Lebugle, Guizar-Sicairos *et al.*, 2019 ▸). The FZP was 200 µm in diameter, had an outermost zone width of 60 nm and was equipped with a 50 µm integrated CS. Scanning is carried out using the combined motion of the sample and a fast FZP scanner, which allows a considerable speed-up of the acquisition (Odstrcil, Lebugle, Lachat *et al.*, 2019 ▸). Diffraction patterns were acquired with an in-vacuum area detector (Eiger 1.5M, pixel size 75 µm) (Dinapoli *et al.*, 2011 ▸; Guizar-Sicairos *et al.*, 2014 ▸) placed 5.237 m downstream of the sample inside an evacuated flight tube.

**Figure 7:**
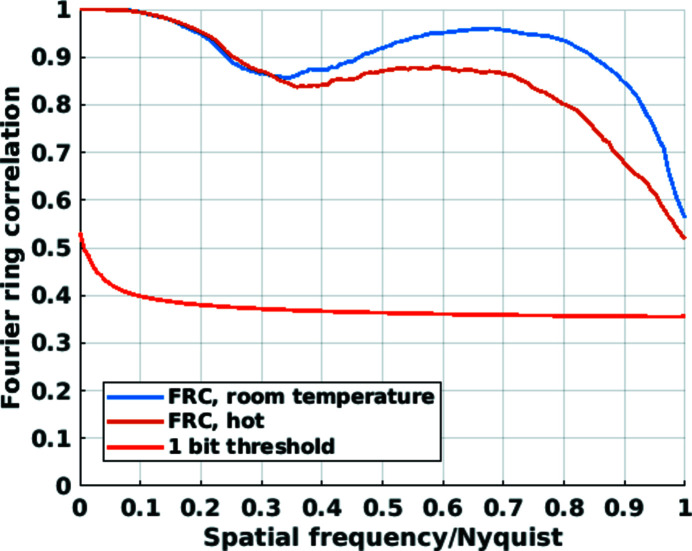
Fourier ring correlation curves of 2D projections of the nanoporous gold sample. Between two projections acquired at a nominal temperature of 50°C (room temperature) and between two projections acquired at a high temperature of 600°C (hot). Pixel size: 19.4 nm.

Each projection consists of multiple diffraction patterns covering a 15 µm × 25 µm (h × v) sample field-of-view (FOV). Scanning was carried out following a Fermat-spiral scanning pattern (Huang *et al.*, 2014 ▸) with an average step size of 1 µm. At each scanning position a diffraction pattern is measured with an exposure time of 50 ms. The total measurement time of a single projection was 34 s. The dimensions of the FOV, in view of the pillar’s diameter and height, is excessive owing to the exploratory nature of the measurements. It later became clear that the instrument drift is sufficiently low to allow for FOVs only slightly larger than the sample size. Ptychography reconstructions were performed using the *PtychoShelves* package (Wakonig *et al.*, 2020 ▸) with 300 iterations of the difference map algorithm (Thibault *et al.*, 2009 ▸) followed by 200 iterations of maximum-likelihood refinement (Thibault & Guizar-Sicairos, 2012 ▸). Due to a partial failure of the detector, a detector area of only 720 × 720 pixels, compared with the otherwise available 1000 × 1000, was utilizable in the ptychographic image reconstruction process. This resulted in a minimum reconstruction pixel size of 19.4 nm at the given detector distance and photon energy. A comparison of Fourier ring correlations (FRCs) (van Heel & Schatz, 2005 ▸) of pairs of projections acquired at different temperatures (50°C versus 600°C) but at the same rotation angle is shown in Fig. 7 ▸. By comparing the curves with the 1-bit threshold curve, we can confirm the high-temperature nano-imaging capability of the introduced system. The half-pitch resolution estimate of projections is, regardless of temperature, 19.4 nm and limited by the minimum pixel size. The area under the correlation curve is reduced for the projections acquired at the higher temperature. Such behavior is expected due to potentially decreased setup stability, but also potential slow sample deformation at higher temperatures. Moreover and observable regardless of temperature is a dip/oscillation in the FRC at about 60 nm. Such oscillations are frequently associated with or caused by features of equivalent size in the sample (Holler, Guizar-Sicairos *et al.*, 2017 ▸).

**Figure 8:**
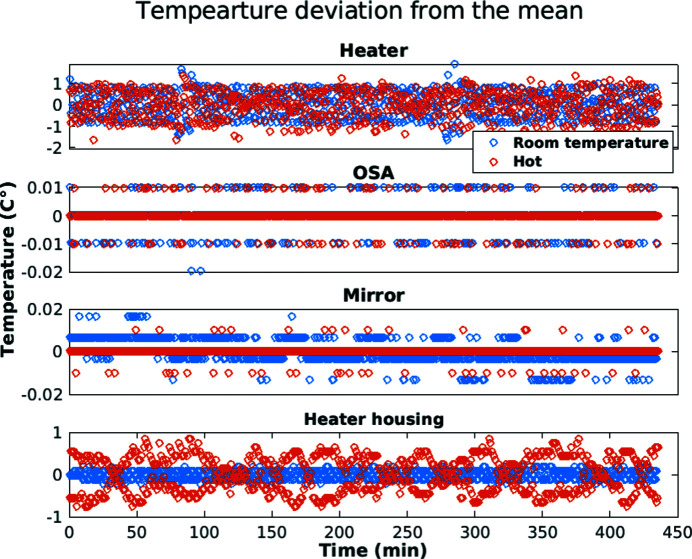
Temperature variations of setup components during tomographic acquisition. The set temperature for the room temperature measurement was 50°C and 600°C for the hot measurement. The mean temperature of the OSA mounting plate was 25.5°C, and the mirror 24.2°C in both cases, while the mean temperature of the heater housing increased from 21°C to 25°C in the hot state.

To facilitate this resolution for nanotomography, the temperature, sample position stability, and image reproducibility need to be maintained over a set angular range and time. To evaluate the system’s performance, 800 angularly equally spaced projections were acquired over 180° at both temperatures. The sample was allowed to stabilize at 600°C for 30 min prior to data collection. The acquisition time per tomogram was 8 h. Fig. 8 ▸ provides temperature deviations of all the relevant setup components during the acquisition of both tomograms. Only minor deviations from the respective set temperatures were registered. It should also be noted that there is no need to realign the OSA when the temperature is changed.

Projections were aligned following the procedures described by Guizar-Sicairos *et al.* (2011 ▸) and Odstrcil, Holler, Raabe & Guizar-Sicairos (2019 ▸). At high temperature the sample experienced slow and small deformations, which were recovered and taken into account using non-rigid tomography (Odstrcil, Holler, Raabe, Sepe *et al.*, 2019 ▸). A maximum deformation of 100 nm was recovered for the high-temperature tomogram.

**Figure 9:**
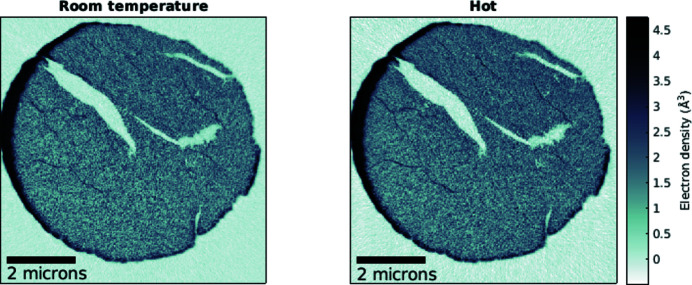
Axial tomographic slices of each measurement. Data displayed on the left were recorded at a set temperature of 50°C, and on the right at 600°C.

**Figure 10:**
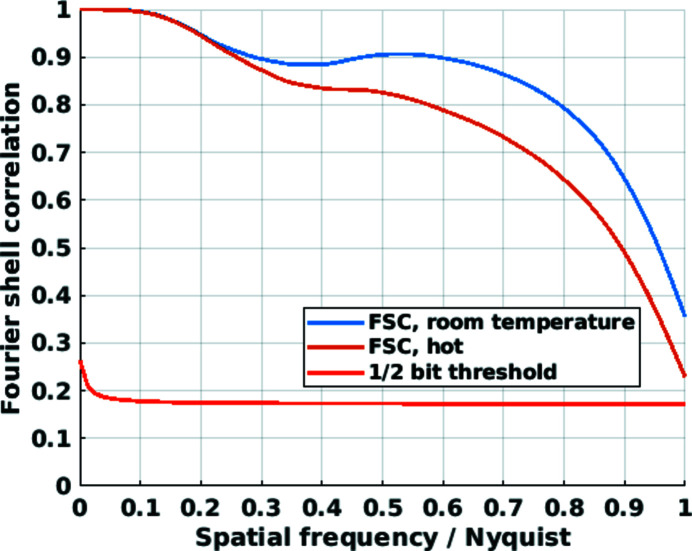
Fourier shell correlation (FSC) of the tomograms of the nanoporous gold sample. The curves suggest a spatial resolution of 19.4 nm, limited by the voxel size, for both the 50°C (room temperature) and 600°C (hot) tomogram.

Axial tomographic slices of the electron-density tomograms (Diaz *et al.*, 2012 ▸) are shown in Fig. 9 ▸. Spatial resolution estimates were obtained using Fourier shell correlation (FSC) analysis for which each dataset was split into two, and independent tomograms were reconstructed and analyzed (Holler *et al.*, 2014 ▸). The resulting curves and resolution estimates are shown in Fig. 10 ▸. The determined half-period resolution estimates are below a single voxel, *i.e.* 19.4 nm, for both tomograms. In agreement with the FRC analysis of projections shown in Fig. 7 ▸, the area under the correlation curve is slightly reduced for the tomogram acquired at the higher temperature.

These measurements confirm that any drifts of the setup during tomogram acquisition are sufficiently small, and demonstrate the capability of high-temperature X-ray nanotomography at variable atmosphere. Further, we found the setup to be stable when ramping the temperature rapidly, even up to nominal sample temperatures of 850°C. As explained above, by design the horizontal drift of the sample in response to temperature changes is expected to be zero. Experimentally we observed a horizontal drift of approximately 1 µm over an 800°C temperature change. In the vertical direction, we expected and observed a shift in sample position between the two tomography measurements, which is caused by the thermal expansion of the tomography pin. This drift required a temperature-dependent adjustment of the height of the sample, which is equivalent to adding a vertical offset to the scanning positions. For the two tomography measurements a correction of up to 40 µm was necessary to retain the same part of the sample within the field-of-view.

To facilitate future dynamic tomography measurements, requiring a systematic or continuous change in temperature, it is important to compensate for these drifts dynamically. We have implemented an image alignment feedback loop for this purpose. This feature uses on-demand ptychographic image reconstructions as well as projection vertical alignment based on vertical mass distribution (Guizar-Sicairos *et al.*, 2011 ▸; Odstrcil, Holler, Raabe & Guizar-Sicairos, 2019 ▸) to compute the required vertical sample shift to maintain the initial field-of-view. This information is continually fed to the measurement control system. Ptychography reconstructions required for this alignment loop do not require the smallest pixel size or highest spatial resolution. Thus, an online reconstruction can be defined such that the time required for the ptychographic projection reconstruction is similar to the acquisition time of a projection. The feedback thus only lacks a few projections behind the measurement.

## 7. Conclusions and outlook

In the presented manuscript, we describe the realization of an environmental control system for X-ray nanotomography. The design of individual hardware components, the control system and the integration into an existing nanopositioning instrument (flOMNI) are detailed. The demonstration of operational capabilities are shown via a comparison of ptychographic tomograms of the same sample acquired at two temperatures (50°C versus 600°C). We demonstrate a 3D half-period resolution of 19.4 nm.

In the future, we envision to use these new capabilities for the realization of *in situ* and *operando* studies of, for example, geological, structural, catalytic, pharmaceutical and energy storage/conversion materials and processes; in particular, those applications that require a controlled atmosphere and elevated temperatures to mimic system-representative environmental conditions and/or extract process dynamics, reaction mechanisms, local compositional changes or phase transformations (Meirer & Weckhuysen, 2018 ▸; Wood, 2018 ▸; Ihli *et al.*, 2021 ▸; Chavez Panduro *et al.*, 2019 ▸; Yasuda *et al.*, 2011 ▸; Cnudde & Boone, 2013 ▸; Marone *et al.*, 2020 ▸). In contrast to the presented, static (instrument capability demonstration) measurements, these dynamic nanotomography measurements, with data acquisition continuing alongside temperature- or atmosphere-induced sample changes, will require new tomography reconstruction and acquisition schemes. The latter can possibly benefit from recent advancements in sparse data acquisition and reconstruction methods as well as non-rigid nanotomography (Gao *et al.*, 2021*a* ▸,*b* ▸; Odstrcil, Holler, Raabe, Sepe *et al.*, 2019 ▸) allowing for the rapid acquisition of hyperdimensional tomograms with increased resistance to sample deformation.

On the instrumentation side, we hope to address two remaining technical difficulties. (1) While the gas temperature is accurately measured, the heat conductivity and resulting temperature gradient from sample to the pin receptor, the latter being close to room temperature, are not well defined. This implies an uncertainty in the precise sample temperature. In a first instance, new OMNY pins with highly reduced heat conductivity compared with the standard metallic ones will be manufactured. The installation of a secondary Bragg diffraction dedicated detector will in the future moreover allow for an online temperature calibration (Parker *et al.*, 2022 ▸). (2) Further, we plan to extend the system’s capabilities to facilitate a controlled operation in a humid environment, for example, to study weathering, condensation and hydration processes on the nanoscale in complex sample geometries. Even further, the construction and installation of a system capable of accommodating liquid samples and able to operate under high-pressure conditions are a future objective.

## Supplementary Material

Section S1: Materials. Figures S1 and S2. DOI: 10.1107/S1600577522006968/ve5158sup1.pdf
