# Supplementary material for: Environmental control for X-ray nanotomography
Source: J Synchrotron Radiat. 2022 Jul 21;29(Pt 5):1223–31. doi: 10.1107/S1600577522006968 (PMC9455200; doi:10.1107/S1600577522006968)
Supplement: Supplementary file 1 [file s-29-01223-sup1.pdf]

JOURNAL OF SYNCHROTRON RADIATION

Volume 29 (2022)

Supporting information for article:

Environmental control for X-ray nanotomography

Mirko Holler, Tomas Aidukas, Lars Heller, Christian Appel, Nicholas W. Phillips, Elisabeth Müller-Gubler, Guizar-Sicairos Manuel, Raabe Jörg and Ihli Johannes

Environmental control for X-ray nano tomography

Mirko Holler, Tomas Aidukas, Lars Heller, Christian Appel, Nicholas W. Phillips, Elisabeth Müller-Gubler, Manuel Guizar-Sicairos, Jörg Raabe, Johannes Ihli

Paul Scherrer Institut, Forschungsstrasse 111, 5232 Villigen PSI, Switzerland

Supporting information

S1. Materials

Silver gold alloy foils (Ag70/Au30) of 250 µm in thickness, were purchased from Goodfellow Cambridge Ltd. The nitric acid solution (69%) was purchased from Sigma-Aldrich.

Nanoporous Gold Sample Preparation: To prepare the nanoporous gold samples, we first mechanically polished the purchased foils to a thickness of ~ 40 µm after which the thinned foil was cut into pieces of ~ 1 mm in diameter. One of the obtained pieces was afterwards submerged in 10 ml of an aqueous nitric acid solution (69%) for 2 hours. The subsequent dealloying process, i.e. the selective dissolution of the more chemically active silver from the alloy by the acid, results in the desired and disordered nanoporous gold structure.(Zinchenko *et al.*, 2013) The sample was then washed first with Milli-Q water and ethanol before being dried at 120 ˚C for 1 hour.

The obtained piece of nanoporous gold was then mounted using epoxy resin on a gold-coated OMNY tomography pin(Holler *et al.*, 2017) and pre-shaped, using a micro-lathe(Holler *et al.*, 2020), into a cylindrical pillar with a diameter of ~30 µm and height of 20 µm. The pillar was next reduced in diameter to roughly 6 µm using focused ion-beam milling, before being transferred to an empty OMNY pin made out of bare aluminum. The transferred pillar was fixed to the pin using platinum deposition.

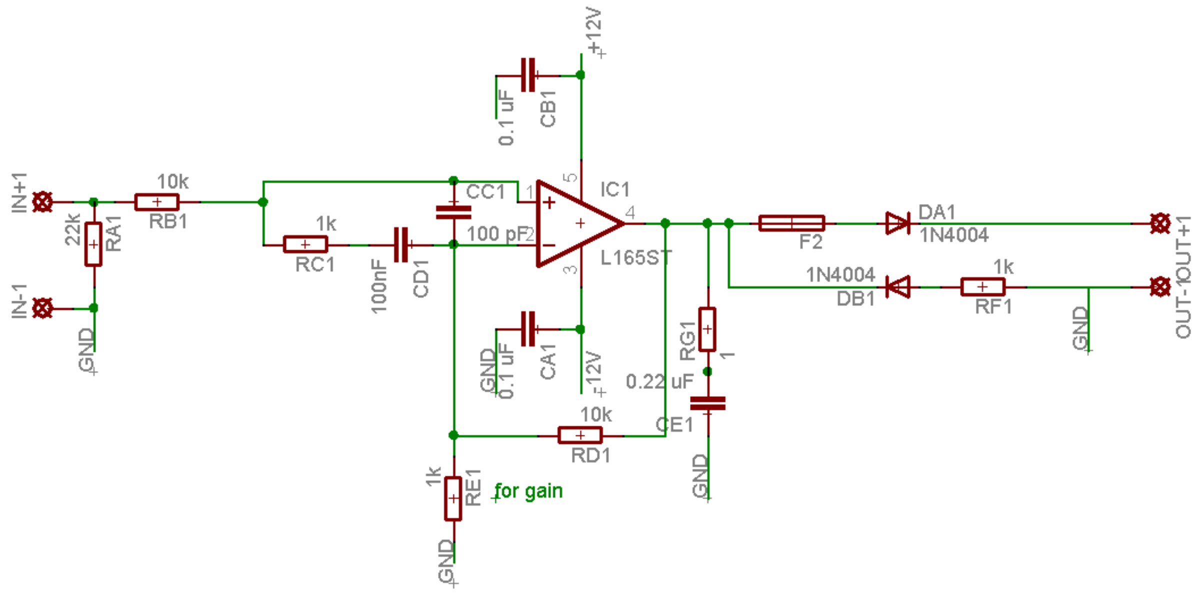

Figure S1 Schematic of one channel of the power operational amplifier circuit used for the low-power heaters at the OSA holder and mirror.

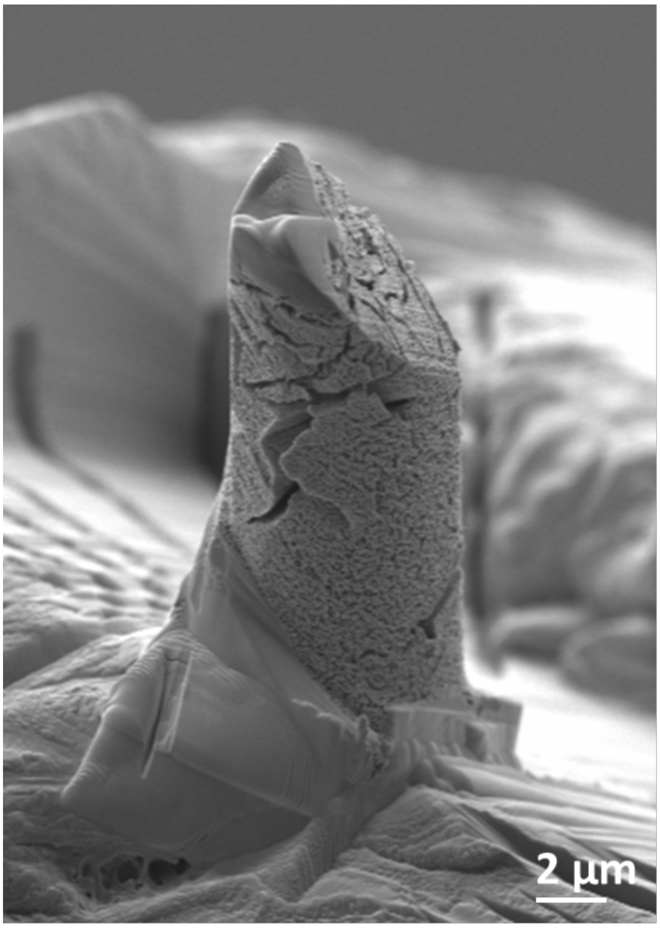

Figure S2 Scanning electron micrograph of a nanoporous gold sample pillar after to lift-out and transfer.

References

Holler, M., Ihli, J., Tsai, E. H. R., Nudelman, F., Verezhak, M., van de Berg, W. D. J. & Shahmoradian, S. H. (2020). *J. Synchrot. Radiat.* **27**, 472-476.

Holler, M., Raabe, J., Wepf, R., Shahmoradian, S. H., Diaz, A., Sarafimov, B., Lachat, T., Walther, H. & Vitins, M. (2017). *Review of Scientific Instruments* **88**, 113701.

Zinchenko, O., De Raedt, H. A., Detsi, E., Onck, P. R. & De Hosson, J. T. M. (2013). *Computer Physics Communications* **184**, 1562-1569.
